# Characteristics of Lumbar Disc Herniation With Exacerbation of Presentation Due to Spinal Manipulative Therapy

**DOI:** 10.1097/MD.0000000000000661

**Published:** 2015-03-27

**Authors:** Sheng-Li Huang, Yan-Xi Liu, Guo-Lian Yuan, Ji Zhang, Hong-Wei Yan

**Affiliations:** From the Department of Orthopaedics (S-LH, H-WY), Central Laboratory for Scientific Research (G-LY), Second Affiliated Hospital, School of Medicine, Xi’an Jiaotong University, Xi’an; Department of Neurosurgery (Y-XL), First Hospital of Yulin City, Yulin; and Department of Orthopaedics (JZ), Weinan Central Hospital, Weinan, China.

## Abstract

The aim of this article was to delineate the characteristics of lumbar disc herniation (LDH) in patients with exacerbation of symptoms caused by spinal manipulative therapy (SMT). The main emphasis should be on the prevention of this condition by identifying relevant risk factors.

Detailed clinico-radiological profiles of a total number of 10 LDH patients with exacerbation of presentation after SMT were reviewed. All the patients underwent neurological and magnetic resonance imaging examinations. Laminectomy and discectomy were performed, and follow-up was carried out in all patients.

The duration of symptoms in the patients before SMT was 4–15 years. After the therapy, an acute exacerbation of back and radicular pain was observed within 24 h. Magnetic resonance imaging showed that L4–L5 was the most frequently affected level observed (7 patients), and each patient had a large disc fragment in the spinal canal. The disc fragments were classified into 3 types according to their localizations. The time internal between the exacerbation of presentation and surgery was 23.1 days. No perioperative complications were noted. All the patients were relieved of radicular pain a few days after surgery. During postoperative follow-up, all patients regained the ability to walk; one patient received catheterization for 1 month and another for 6 months. Eight patients reported a complete resolution of presentation and the rest 2 patients were significantly improved.

SMT should be prohibited in some LDH patients to prevent neurological damages, in whom there are 5 possible risk factors. Surgical results for these patients are encouraging.

## INTRODUCTION

The management of lumbar disc herniation (LDH) most commonly involves conservative measures such as bed rest, bracing, traction, and spinal manipulative therapy (SMT). The great majority of LDH patients got satisfactory recovery through noninvasive therapy.^1,2^ In China, the most prevalent therapy for LDH is SMT, a manual procedure practiced by chiropractors for the management of this disease.

There are, however, some patients, small in number but of great importance, in whom SMT aggravates symptoms and operative treatment is imperative. Oliphant^3^ found that the incidence of LDH exacerbation following SMT is 1:3.72 million. The disease process worsens if surgical treatment is not performed. Some studies suggested that SMT might be in relation to the deterioration of the clinical symptoms.^4–6^ Unfortunately, there have been few articles in the literature discussing the potential risk factors for LDH patients to be treated with SMT. Even though the aggravated symptoms due to SMT are relatively rare in patients with LDH, they can potentially cause severe neurological symptoms and disabilities. In addition, aggravation of symptoms caused by medical mismanagement often results in litigation.

In LDH patients, the aggravated symptoms due to SMT are profound clinical problems and have remained challenging to chiropractors. Safety concerns of SMT for LDH patients have become important topics. Significantly worsened signs in such patients warrant further investigation. The clinical characteristics should be identified. The purpose of this study was to analyze the risk factors that cause deterioration of clinical symptoms so as to help chiropractors better avoid malpractice when treating patients with low back pain. We reviewed 10 LDH patients who experienced aggravated symptoms due to SMT and then received surgical treatment, and analyzed the clinical characteristics and outcomes in these patients.

## MATERIALS AND METHODS

The study protocol was approved by the Ethics Committee of the Second Affiliated Hospital, School of Medicine, Xi’an Jiaotong University, Xi’an, China. Written informed consent was obtained from all the patients included in this study.

Between January 2011 and December 2012, 10 consecutive patients with exacerbation of LDH caused by SMT were treated at our hospital. There were 5 men and 5 women ranging from 46 to 68 years, with an average age of 60.5. It is striking that 70% of the patients were older than 50 years. Among these 10 patients, 4 had associated hypertension. All the patients complained of back pain for varying durations from 4 to over 10 years before SMT; 9 patients had very long history of symptoms, perhaps more than 5 years.

All the patients had received manipulative treatment on the lumbar spine under consciousness before admission, but the information regarding the accurate manipulative techniques was not obtained. There was no history of trauma or back surgery in any of these patients. The diagnosis was based on magnetic resonance imaging (MRI) and objective clinical findings. The affected vertebral level was determined using MRI. The medical charts were reviewed, which included age, sex, presenting symptoms, neurological status, radiological findings, operative findings, and treatment results. The detailed information of the patients is presented in Table 1.

**TABLE 1:**
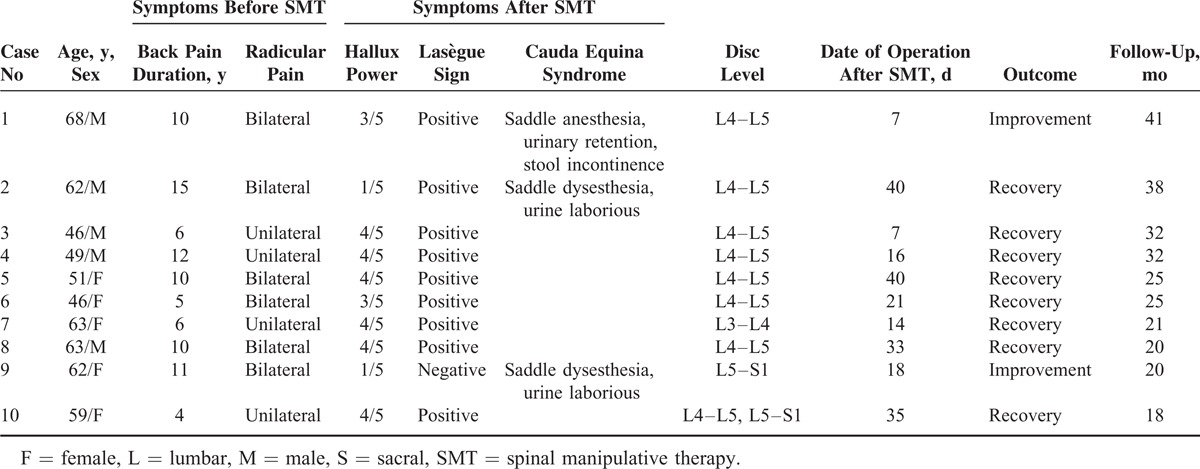
Summary of Clinical Findings in 10 Patients With Lumbar Disk Herniation

In all these patients, conservative treatment consisted of bed rest and the use of nonsteroidal anti-inflammatory drugs could not improve symptoms, and surgical intervention was thus imperative. Excision of the disc through posterior approach via laminectomy was performed in all the patients. Intervertebral disc space levels were reconfirmed using image intensifier. After the removal of the free fragments, the remaining material in the disc space was also removed. The outcome of surgery was assessed during the follow-up period in terms of improvement in sensory, power, and sphincter function.

## RESULTS

### Clinical Features

In this series, aggravated symptoms post manipulation included local back pain, leg pain, and/or paresthesia. Four patients developed additional neurological deficits after SMT. These signs included foot drop, saddle anesthesia, and urinary incontinence, which occurred between 0 hour and 24 hours after SMT. Lower back and radicular pain were present in all the patients, and the pain became worse as time went on. Subjective numbness and weakness of feet and toes were also reported. Initial clinical examination revealed restriction of back movements in all the patients. Nine patients had lower back pain for more than 5 years. Pain affected both lower limbs in 6 patients, often with 1 leg worse than the other.

At admission, all patients had trouble walking and standing in an upright position, and almost could not sit or even lie down. The straight leg raise (Lasègue test) was positive in most patients and was less than 45° in various patients. These symptoms indicated a severe disability caused by pain. One patient with negative straight leg raise had more intense pain in a supine position than in a sitting position, so she slept only in a sitting position. Two patients had hesitancy of micturition, but they were continent, without catheters or pads. Only 1 patient was catheterized at the time of admission.

### Radiographic Findings

Of these 10 patients, 9 (90%) were admitted to our hospital with diagnostic images from other hospitals. All patients had a large lumbar disc herniation demonstrated by MRI, on which massive disc herniation occupied more than one third of the canal diameter. On T1-weighted images the prolapsed disc appeared iso or hypointense, whereas on T2-weighted images the lesion appeared hypo or hyperintense. The level of the herniation was L4–L5 in 7 patients, and was L3–L4, L5–S1, and L4–S1 in the rest 3 patients, respectively. There was a general consistency between the level of disc herniation in MRI and the clinical signs, symptoms, and examination findings.

**FIGURE 1:**
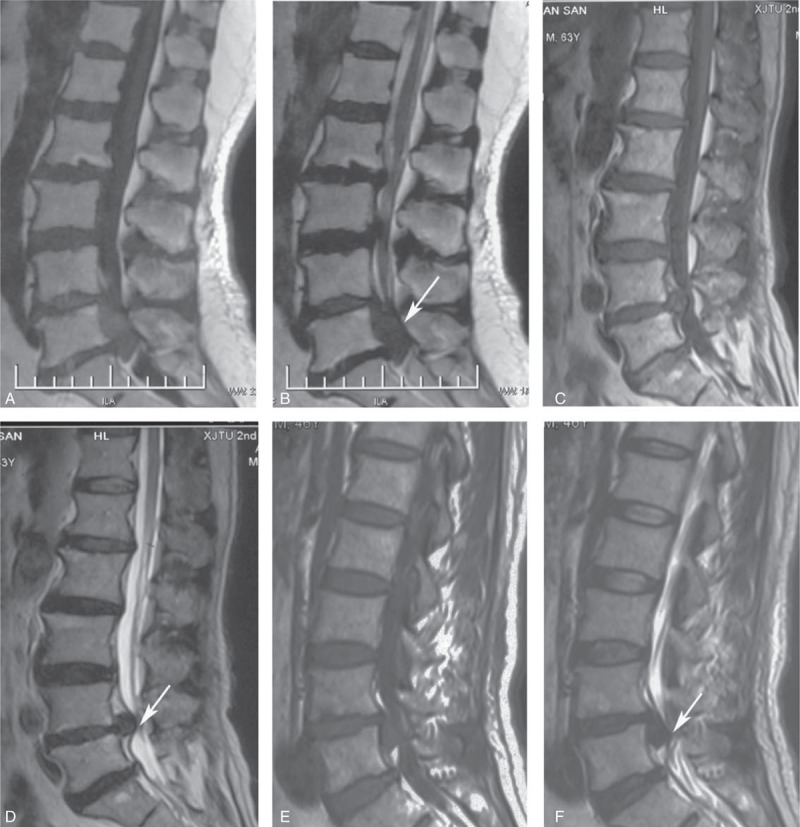
MRI showing the location of the disc fragments. (A, B) Cephalic type. (C, D) Ventral type. (E, F) Caudal type. (A, C, E) T1-weighted images. (B, D, F) T2-weighted images.

The morphological characteristics of the affected disc level were determined on the basis of MRI findings. Anatomically, the disc material is commonly located at the ventral part of the spinal canal. Disc fragments may migrate within the spinal canal in superior, inferior, and posterior directions to the anterior epidural space. On the basis of an upward or downward migration of the disc fragment derived from the adjacent level, the location of a disc fragment should be described in the cranio–caudal plane as either cephalic type (supradisc level), caudal type (infradisc level), or ventral type (at disc level) (Figure 1). Cephalic type refers to the condition that a disc fragment locates behind the upper vertebral body of the corresponding intervertebral disc level; caudal type refers to the situation that a disc fragment locates behind the lower vertebral body of the corresponding intervertebral disc level; and ventral type refers to the case that a disc fragment stays in the level of the disc. In our patients, cephalic type was found in 2 patients, caudal type in 3 patients, and ventral type in 5 patients.

### Operative Findings

The mean time from presentation to surgery in the 10 patients was 23.1 days (range from 7 to 40 days). But only 2 patients were operated on within 2 weeks after SMT. Rupture at the posterior longitudinal ligament and anulus was detected during operation. Single-level discectomy was performed in 9 patients and 2-level laminectomy was performed in 1 patient. There were no operative deaths. No deterioration in the neurological status was observed in any of the patients in the immediate postoperative period, and no postoperative complications occurred. Complete pain relief was observed in all the patients within the first few days after the operation.

### Histopathology

Biopsy revealed that the excited parts were disc material. The histopathological diagnosis was consistent with a degenerated intervertebral disc.

### Follow-Up

The follow-up period ranged from 18 to 41 months. Two patients continued using Foley catheters after operation. One was able to have the catheter removed after 1 month and the other after 6 months. During the follow-up period, no patient suffered any recurrent back pain or other adverse effects. At the last follow-up, all the patients were ambulatory. Eight patients were almost normal; 2 patients had leg weakness and used canes to help them walk.

## DISCUSSION

There is controversy regarding the effects of SMT on LDH. Evidence showed that SMT has a beneficial effect on pain, straight-leg raising, range of motion, size of disk herniation, neurological symptoms, whereas other data showed that SMT is responsible for causing LDH and cauda equine syndrome.^3^ Although less frequent, deterioration of the LDH presentation that occurs after SMT is of the utmost importance because it represents potentially avoidable lesions. The presentation of LDH may differ in each individual patient. In general, this disease is characterized by low back pain, unilateral or bilateral sciatica, motor weakness of lower extremities, and deep tendon reflexes abnormalities. The symptoms and signs may be observed asymmetrically and incompletely in patients. After manipulative treatment, the back pain and sciatica increase severely and suddenly, and there is often motor weakness. These symptoms usually develop in less than 24 hours. The most common sensory dysfunction is reduction in pin prick and light touch. Motor weakness at varying degrees in the lower limbs is a key component in the diagnosis. Bilateral symptoms and signs commonly indicate the imminence. Depending on the level of the disc herniation, varying reflex losses can be observed. The leading symptom of all our patients was pain in the lower back and leg (s), and the pain was severe, indicating an acute and extensive compression of spinal roots. All patients showed weakness in the legs affecting walking or standing, which indicated compression of the spinal cord or cauda equine. In addition, the patients with symptoms aggravated by SMT predominated in their fifth or sixth decade of life, which was older than the overall population with LDH.

MRI, as a noninvasive radiological investigation, is regarded as the most reliable method for diagnosing LDH and is also of crucial importance in guiding the management of LDH. It can also detect any concomitant spinal cord anomalies.^7–14^ MRI should be assessed in all patients who present new onset of symptoms or abrupt, more severe symptoms, and signs in the context of SMT to identify the extent of LDH and assess the prognosis. Urgent MRI is especially recommended in patients with sudden aggravated or new onset of symptoms after SMT. MRI reveals that the disc fragment is central or paramedian, and the commonest level involved is L4–L5. Sagittal sections are extremely useful in delineating the extent of the migration of the disc material behind the vertebral body.

LDH is among the most common claims against chiropractors. The manipulative techniques used vary from one chiropractor to another. Standardization of SMT regarding LDH is difficult. There is evidence that SMT as a therapeutic procedure seems to be risky.^4,15,16^ In China, the decision to seek SMT depends more on the patients’ own perceptions. These patients frequently prefer to accept SMT rather than other therapies on this entity because they are unaware of the risk that SMT could cause significantly worsened LDH, even cauda equina syndrome. Therefore, it is necessary to estimate the risk factors of SMT in treating LDH. All chiropractors who deal with spinal diseases should be aware of the possibility of aggravated symptoms of LDH after SMT, and the most important issue is the identification of high-risk patients.

Although symptoms and signs of LDH may be variable, they can usually provide vital clues to chiropractors. On the basis of the clinical features of LDH and our experience in managing the lesion, we found that most patients, presented with LDH exacerbation following SMT, had the following characteristics: older than 50-year old; repeated episodes of lower back pain with alternating sciatica; long-standing lower back pain and sciatica over a period of 5 years; MRI-documented severe disc herniation; and bilateral symptoms and signs. Therefore, SMT should not be recommended to patients with 1 or more of these conditions. In our experience, these symptoms could be harbingers of medico-legal actions against chiropractors. There was predominance in the patients who are 50 years or older. Because manipulation is not contraindicated in patients with LDH,^17^ we suggest that the above listed 5 conditions be risk factors for the adoption of SMT in treating LDH. Chiropractors should be aware of these factors so as to reduce the incidence of aggravation. Whenever there are preexisting symptoms suggesting risk factors of vigorous SMT, manipulation of the lumbar spine should not be performed.

The pathophysiological mechanisms for the SMT-induced aggravation of symptoms in LDH are not completely understood. The only loading conditions, which involve a combination of compression, lateral bending, and forward bending, cause posterior disk prolapse.^3,18,19^ Although the standard lumbar spinal manipulation in the side position does not involve these movements, variation in patient positioning and load application with various maneuvers may yield different results.^20^ If the axial rotation of the lower lumbar vertebrae is great enough, the annulus can be injured with rotation.^21^ In addition, disc migration caused by SMT might accentuate the compression of the disc on the affected nerve roots at the corresponding level, causing edema of the roots and thus accentuating the neurological lesion. We have found in our patients the compression on the nerve roots is not promptly and effectively relieved unless surgery is performed. Conservative management oftentimes cannot resolve the problems. The outcome of surgery is favorable in most patients. Especially, it provides relief of back pain. Most of our patients did not have prompt decompressive surgery. There was an appreciable delay between the onset of symptoms and surgery in the patients due to their late presentation to the hospitals, which could be partly attributed to their poverty and apprehension of the surgical complication. Delayed surgical decompression might not materially affect outcomes. It is well known that optimal timing of surgery is unknown. A report claimed no difference in the outcome between early and late operations after the onset of symptoms,^22^ and the experiment on an animal model found no significant difference in the degree of recovery following immediate, early, and late surgical decompression to the cauda equina.^23^ Despite late intervention in our patients, we did find superior outcomes. We believe that the timing of surgery was less important than the severity of increasing symptoms. Although results of surgery were difficult to compare because of variability in symptoms, all patients improved.

We find that peripheral motor deficits recover more quickly after operation compared with urological dysfunction. Recovery of bladder function is usually slow. Postoperative result is very good regarding the relief of pain. The motor recovery is better than the sensory recovery and all the patients are walking again; this is because the motor nerves recover faster than the sensory nerves with constriction removed.^24,25^ All patients, irrespective of whether they were operated early or late after the aggravation of presentation, have good power of lower extremities.

This study had the following limitations. The series of patients involved was small, which caused difficulty in analysis and made it hard to reach definite conclusions. Further study with larger series might help elucidate this uncommon lesion. In addition, the chiropractors’ techniques were not obtained, and it is possible that harsh technique and excessive lumbar flexion during rotational manipulation might contribute to the exacerbation of the symptoms in these patients. And chiropractors differed in training, experience, and type of practice, which led to varied conditions in patients.

## CONCLUSION

In this study, we reviewed 10 patients of LDH, who underwent surgical treatment due to exacerbation of presentation caused by SMT. Five risk factors have been identified regarding the treatment of LDH by SMT. The present data attempt to offer guidance to chiropractors for the appropriate management of patients. Chiropractors should assess patients with back pain before performing SMT and practice the manipulation particularly carefully if any of the risk factors exists. To the best of our knowledge, this is the first study to address the risk factors of SMT in the treatment of LDH.
